# An IFN-STAT Axis Augments Tissue Damage and Inflammation in a Mouse Model of Crohn's Disease

**DOI:** 10.3389/fmed.2021.644244

**Published:** 2021-05-20

**Authors:** Iris Stolzer, Anja Dressel, Mircea T. Chiriac, Markus F. Neurath, Claudia Günther

**Affiliations:** ^1^Medizinische Klinik 1, Universitäts-Klinikum Erlangen, Friedrich-Alexander-Universität Erlangen-Nürnberg, Erlangen, Germany; ^2^Deutsches Zentrum Immuntherapie, Universitäts-Klinikum Erlangen, Friedrich-Alexander-Universität Erlangen-Nürnberg, Erlangen, Germany

**Keywords:** inflammatory bowel disease, STAT signaling, inflammation, cell death, Paneth cells

## Abstract

Blocking interferon-function by therapeutic intervention of the JAK-STAT-axis is a novel promising treatment option for inflammatory bowel disease (IBD). Although JAK inhibitors have proven efficacy in patients with active ulcerative colitis (UC), they failed to induce clinical remission in patients with Crohn's disease (CD). This finding strongly implicates a differential contribution of JAK signaling in both entities. Here, we dissected the contribution of different STAT members downstream of JAK to inflammation and barrier dysfunction in a mouse model of Crohn's disease like ileitis and colitis (*Casp8*^Δ*IEC*^ mice). Deletion of STAT1 in *Casp8*^Δ*IEC*^ mice was associated with reduced cell death and a partial rescue of Paneth cell function in the small intestine. Likewise, organoids derived from the small intestine of these mice were less sensitive to cell death triggered by IBD-key cytokines such as TNFα or IFNs. Further functional *in vitro* and *in vivo* analyses revealed the impairment of MLKL-mediated necrosis as a result of deficient STAT1 function, which was in turn associated with improved cell survival. However, a decrease in inflammatory cell death was still associated with mild inflammation in the small intestine. The impact of STAT1 signaling on gastrointestinal inflammation dependent on the localization of inflammation, as STAT1 is essential for intestinal epithelial cell death regulation in the small intestine, whereas it is not the key factor for intestinal epithelial cell death in the context of colitis. Of note, additional deletion of STAT2 was not sufficient to restore Paneth cell function but strongly ameliorated ileitis. In summary, we provide here compelling molecular evidence that STAT1 and STAT2, both contribute to intestinal homeostasis, but have non-redundant functions. Our results further demonstrate that STATs individually affect the distinct pathophysiology of inflammation in the ileum and colon, respectively, which might explain the diverse outcome of JAK inhibitors on inflammatory bowel diseases.

## Introduction

Based on similarity in their structure and function as well as sharing of downstream signaling pathways, interferons (IFNs) are grouped into three families: Type I with IFNα, IFNβ, and several minor subtypes; Type II with IFNγ and Type III with IFNλs. Their expression can be induced in response to diverse viral and bacterial stimuli in an autocrine or paracrine fashion (1–3). Ligation of IFNs to their corresponding receptors activates the Janus kinase (JAK)-signaling transducer and activator of transcription (STAT) signaling pathway. Activated JAKs induces STAT dimer formation, homo- or heterodimer depending on the context, and subsequently expression of different target genes involved in various biological processes (2, 4, 5). The canonical JAK-STAT signaling includes STAT1-STAT2 heterodimers, with associated complex formation, in response to type-I and type-III interferons and STAT1-homodimers following IFNγ ligation. However, non-canonical JAK-STAT signaling in response to all types of IFNs can provoke homodimer formation (all STATs 1–6) as well as different combinations of heterodimers with other members. Beside this, type-I IFNs can also induce gene activation independently of STATs. This manifold setting, with various dimer and complex formations, enables the modulation of gene transcription of several hundred different genes by IFNs (2–6). Accordingly, IFNs are currently considered as key cytokines in autoimmune diseases such as inflammatory bowel disease (IBD), rheumatoid arthritis or autoimmune hepatitis (7–12). Inflammatory bowel diseases are prototypic immune-mediated inflammatory diseases with a globally increasing prevalence, affecting the gastrointestinal tract. IBD includes the major forms ulcerative colitis and Crohn's disease. The main differences of these both forms is the localization of inflammation: While ulcerative colitis only affects the colon, Crohn's disease can cause inflammatory lesions along the whole gastrointestinal tract predominantly in the terminal ileum. In this context, it has just recently been shown that IFNλ is a key factor for small intestinal inflammation that can trigger mucosal inflammation by influencing host cell death pathways in the context of IBD (13). Crohn's diseases patients displayed increased IFNλ serum and tissue levels associated with severe inflammation, and increased cell death accompanied by a dramatic reduction of Paneth cell numbers in the small intestine. The same study depicted that interferons were able to alter *Caspase-8* as well as *Mlkl* gene expression in intestinal epithelial cells (IECs) to induce apoptosis and necroptosis (13). In contrast to this, IFNλ promotes tissue regeneration and mucosal healing in the colonic tissue, highlighting a specific regulation of downstream signaling mechanisms depending on the cellular and spatial context (14).

Caspase-8 is a central cell death regulator that is involved in various cellular processes. Translational studies have demonstrated that humans with Caspase-8 mutations display an early onset IBD associated with massive epithelial cell death and severe inflammation (15, 16). In line with these studies it has been previously shown that mice lacking this central cell death regulator (*Casp8*^Δ*IEC*^) in intestinal epithelial cells spontaneously develop intestinal inflammation with histomorphological alterations similar to the classical features of Crohn's disease. Accordingly, *Casp8*^Δ*IEC*^ mice mimic several important characteristics including Paneth cell depletion accompanied by microbial dysbiosis, the culprit of inflammation in the ileum, as well as the immune cell signature (Th1 driven) association with elevated IFN levels (17–19). Inflammation in these mice is primarily located in the distal part of the small intestine (ileum), and depending on the microbial composition can vary in extent and localization to cause colitis or extensive enteritis (17, 18). Colonic inflammation is dependent on TNFα signaling, whereas TNFα deficiency is able to ameliorate colonic inflammation, but is not sufficient to prevent Paneth cell loss or enteritis in the small intestine (19, 20). Hence, intestinal epithelial cell death seems to have shared general mechanism but requires a tissue specific regulation.

Emerging evidence have indicated IFNs and their immune-modulatory function, as well as their impact on cell death mechanisms, as important factors in the pathogenesis of IBD and as a central point for therapeutic intervention (13, 21). Current strategies focus on blocking the JAK-STAT signaling downstream of IFNs. Tofacitinib, a small molecule JAK inhibitor, can attenuate disease activity in patients with active ulcerative colitis accompanied by improved clinical response and mucosal healing (22, 23). While these data are promising, tofacitinib was insufficient to induce a clinical benefit for patients with Crohn's disease (24–26). Phase II trials reported biological activity of tofacitinib in Crohn's disease patients, but without a significant clinical benefit (24–26) and even indicated disease worsening (25). In line with this, recent data derived from preclinical studies, demonstrated that intestinal inflammation in CD and UC models displayed different disease mechanisms associated to IFN-coordinated cell death (13, 14). In contrast to the broad JAK inhibitor tofacitinib (JAK1 and JAK3 inhibitor), more selective inhibitors like filgotinib and upadacitinib (JAK1 inhibitor) seem to have more therapeutic benefit in both diseases but clinical trials are currently ongoing. For Crohn's disease patients, filgotinib reduced fecal calprotectin and C-reactive protein levels, and was associated with mucosal healing and clinical remission (27). However, broad JAK inhibitors such as tofacitinib not only block IFN-signaling. Depending on the cellular context, various cytokines can activate the JAK-STAT signaling pathway, e.g., IL-6, IL-10, which can result in the activation of various STAT pathways (28). Clinical trials using either specific or broad JAK-inhibitors, reported differences in clinical benefit and treatment success which might be explained with the differential role and relevance of the underlying signaling cascade depending on JAK-STAT pathway. Accordingly, detailed mechanisms are still missing and further knowledge is required.

Here we provide molecular evidence that STAT1 and STAT2 both contribute to intestinal inflammation but have non-redundant functions. Of note, the impact of STAT1 signaling on intestinal inflammation seems to be strongly dependent on the localization of inflammation as STAT1 is involved in cell death regulation in the small intestine associated with Paneth cell death, whereas it is not the key factor for epithelial death in the context of colitis.

## Materials and Methods

### Mice

*Casp8*^Δ*IEC*^ (17), *Casp8*^Δ*IEC*^x*Mlkl*^−/−^ (13), *Stat1*^−/−^ (29), *Stat2*^−/−^ (30) mice were described earlier. *Casp8*^Δ*IEC*^x*Stat1*^−/−^ were generated by crossing *Casp8*^Δ*IEC*^ mice to *Stat1*^−/−^ mice, *Casp8*^Δ*IEC*^x*Stat1*^+/−^x*Stat2*^−/−^ and *Casp8*^Δ*IEC*^x*Stat1*^−/−^x*Stat2*^−/−^ mice were generated by crossing *Casp8*^Δ*IEC*^ mice to *Stat1*^−/−^x*Stat2*^−/−^ mice. As controls we used littermates or C57BL/6 mice. At the end of the experiments, mice were sacrificed by cervical dislocation. Mice were routinely screened for pathogens according to FELASA guidelines. Animal procedures were approved by the Institutional Animal Care and Use Committee of the Regierung von Unterfranken and conducted by qualified personnel.

### DSS-Colitis

Experimental colitis was induced in mice by the administration of 2% dextran sodium sulfate (DSS) in the drinking water for 5 days. The development of colitis was monitored with a high-resolution video mini-endoscopic system. Endoscopic scores for intestinal inflammation were assigned based on the criteria described for the assessment of the murine endoscopic index of colitis severity, which scores the following parameters: translucency, granularity, fibrin, vascularity, and stool consistency, as previously described (31).

### Organ Collection and Storage

Tissue for histology and immunohistochemistry were collected and fixed in 4.5% PFA. Tissue was embedded in paraffin in a water-free procedure and stored at room temperature for further analysis. Samples for RNA and protein analyses were instantly frozen in liquid nitrogen and stored at −80 °C until further use.

### Histology and Immunohistochemistry

Histopathological analyses were performed on formalin-fixed paraffin-embedded tissue cross sections after Mayer's haematoxylin and eosin (H&E) staining or a combined staining with periodic acid–Schiff reaction and alcian blue (PAS). Immunofluorescence of tissue sections was performed using Streptavidin Protein DyLight (Thermo). Primary antibodies (for detailed information see Supplementary Table 1) were incubated overnight. Nuclei were counterstained with Hoechst 33,342 (Invitrogen). Cell death (TUNEL) was analyzed using the *In-Situ* Cell Death Detection Kit (Roche). Images were obtained using the microscope LEICA DMI 4000B together with the LEICA DFC360 FX or LEICA DFC420 C camera or the microscope Leica DMi1 with the corresponding imaging software.

### Gene Expression Analysis

Total RNA was extracted from intestinal tissue using the peqGOLD Total RNA Kit or Total peqGOLD Microspin Total RNA Kit for organoids (peqLab/VWR). cDNA was synthesized by reverse transcription using the SCRIPT cDNA Synthesis Kit (Jena Bioscience) and analyzed by real-time qPCR using SYBRGreen reagent (Roche), the LightCycler 480 (Roche) and specific QuantiTect Primer Assays (Qiagen) (for detailed information see Supplementary Table 2). Experimental values were normalized to levels of the housekeeping gene *hypoxanthine guanine phosphoribosyl transferase* (*Hprt*) or *Glyceraldehyde 3-phosphate dehydrogenase* (*Gapdh*). For fold change calculation, the average mean of the relative expression of control mice were set as 1.

### Organoid Culture

Small intestinal organoids were isolated from the mouse small intestine and cultured with ENR medium (organoid medium with epidermal growth factor/Noggin/R-spondin) for a minimum of 7 days according to Sato et al. (32). Organoid growth was monitored by light microscopy. Organoids were stimulated with TNFα (25 ng/ml) and IFNβ (100 ng/ml) and stained with Propidium Iodide Staining Solution (BD Pharming). Images were obtained using the microscope LEICA DMI 4,000 B together with the LEICA DFC360 FX or LEICA DFC420 C camera or the microscope Leica DMi1 with the corresponding imaging software.

### Statistical Analyses

Comparisons of two groups were performed using an unpaired two-tailed *t*-test. Comparisons among multiple groups were performed using ANOVA followed by multiple comparison and statistical significance was accepted with *p* < 0.05 (NS *p* ≥ 0.05; ^*^p < 0.05; ^**^p < 0.01; ^***^p < 0.001; ^****^p < 0.0001). Statistical calculations were performed using GraphPad Prism 8 (GraphPad Software).

## Results

STAT1 signaling attenuates MLKL mediated necroptosis in the ileum and partially restores Paneth cell function.

Previously, we have demonstrated that IFNs can trigger programmed necrosis by regulating *Mlkl* gene transcription via activation of the transcription factor STAT1 in hepatocytes (33). Furthermore, STAT1 alters the gene expression of *Caspase-8* and *Mlkl* in the small intestine and interferon-induced cell death seems to plays a crucial role during Crohn's disease like ileitis (13). To study the impact of STAT1 on gastrointestinal inflammation with features of Crohn's disease, we deleted this transcription factor in the *Caspase-8* mouse model (*Casp8*^Δ*IEC*^). Generation of double deficient mice strain (*Casp8*^Δ*IEC*^x*Stat1*^−/−^) was not trivial, as both genes are closely located on chromosome 1 (*Caspase-8*: 1 C1.3; 1 29.19 cM, *Stat1*: 1 C1.1; 1 26.81 cM), suggesting that STAT1 might be relevant upstream of Caspase-8.

**Figure 1:**
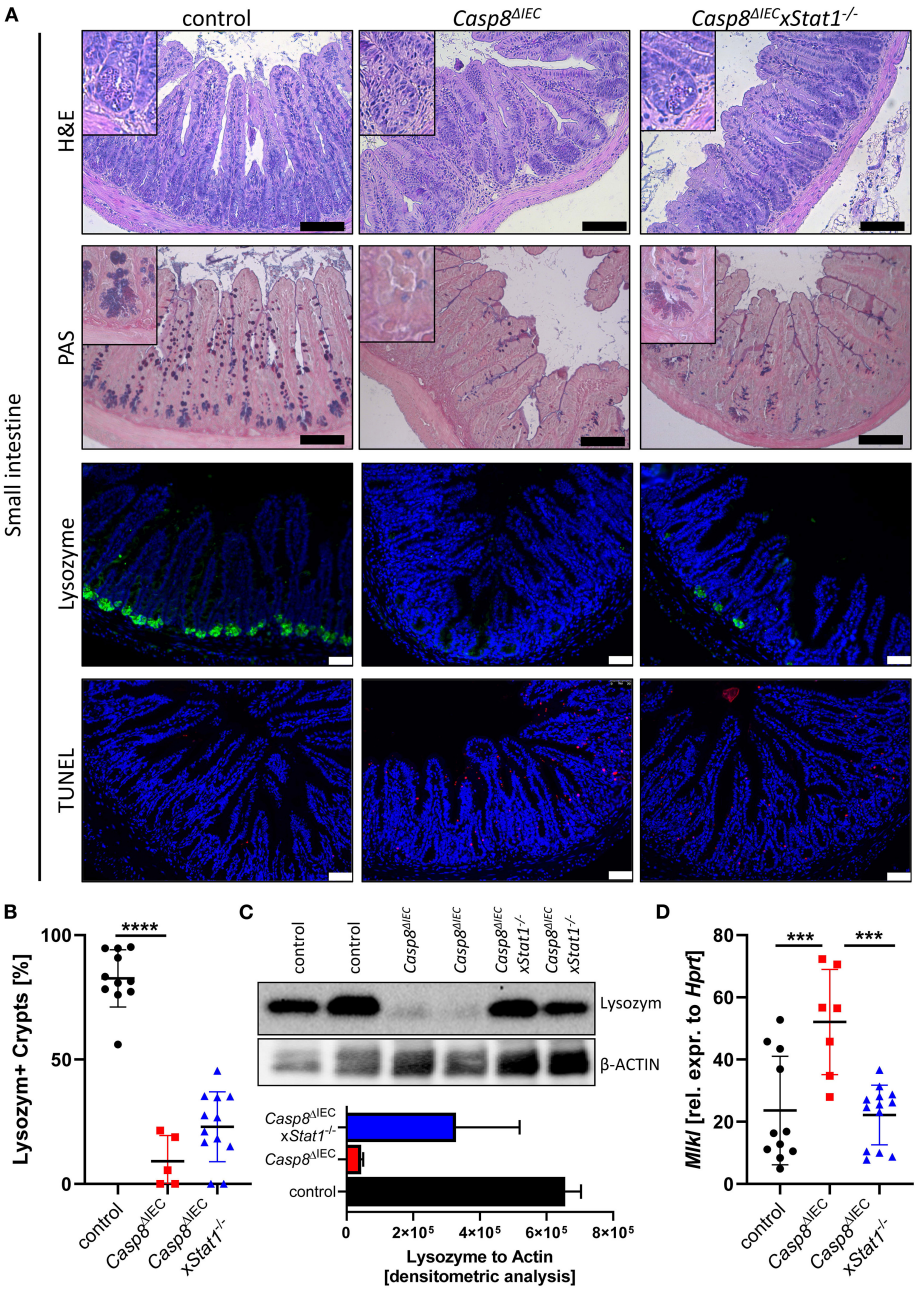
STAT1 signaling attenuates necroptosis and partially restores Paneth cell depletion. **(A)** Representative images of small intestinal tissue cross sections of wild type control, *Casp8*^Δ*IEC*^, and *Casp8*^Δ*IEC*^xStat1^−/−^ mice stained with standard H&E, PAS (scale bar: 100 μm) or immunohistochemically stained (scale bar: 50 μm) with antibody against Lysozyme (green) or stained with TUNEL assay (red). Nuclei were counterstained with Hoechst 33,342 (blue). **(B)** Quantification of Lysozyme positive crypts. **(C)** Western blot analysis Lysozyme in murine ileal tissue. β-Actin was used as loading control. Densitometry analysis for quantification (*n* = 2). Error bars i1ndicate +SD. **(D)** Gene transcription analysis of *Mlkl* mRNA expression in small intestinal tissue. *Hprt* was used as housekeeping gene. Error bars indicate +/−SD Statistical analyses: One-way ANOVA with Tukey's multiple comparisons test; NS *p* ≥ 0.05; *p < 0.05; **p < 0.01; ***p < 0.001; ****p < 0.0001.

*Casp8*^Δ*IEC*^ mice are characterized by MLKL mediated epithelial necroptosis, Paneth cell depletion and ileitis (17). In line with these observations, gene deletion of *Mlkl* in *Casp8*^Δ*IEC*^ mice (*Casp8*^Δ*IEC*^x*Mlkl*^−/−^ mice) was sufficient to block Paneth cell death and inflammation (17) (Supplementary Figure 1). These data clearly demonstrate that indeed Caspase-8 is a negative regulator of MLKL mediated cell death in the intestinal epithelium. In line with our previous observation, mice lacking Caspase-8 displayed rare to no Paneth cells as visualized by PAS staining or specific Lysozyme detection (Figure 1A). However, deletion of *Stat1* in *Casp8*^Δ*IEC*^ mice partially restored Paneth cell numbers as visualized by quantification of crypts containing Lysozyme^+^ cells (Figure 1B). Accordingly, restriction of IFN-STAT1 pathway increase the ratio from around 5 % Paneth cells in *Casp8*^Δ*IEC*^ mice up to 25 % in *Casp8*^Δ*IEC*^x*Stat1*^−/−^ mice (Figures 1A,B). This could be confirmed by quantification of Lysozyme production by Western Blot (Figure 1C). Previously, we have demonstrated that Paneth cell depletion in a Caspase-8 proficient context is associated with IFN-induced cell death and STAT1 mediated transcriptional control of *Mlkl* gene expression (13). In line with these previous data, STAT1 deletion in *Casp8*^Δ*IEC*^ mice was associated with decreased *Mlkl* gene expression and a reduced number of TUNEL (terminal deoxynucleotidyl transferase dUTP nick end labeling) positive dying cells (Figures 1A,D). However, Paneth cell function could not be restored to normal. Furthermore, staining of the Leukocyte Common Antigen (LCA, CD45), displayed higher leucocyte infiltration, especially at the crypt button in *Casp8*^Δ*IEC*^ mice but also in *Casp8*^Δ*IEC*^x*Stat1*^−/−^ mice compared to control mice (Supplementary Figure 2A). Hence, STAT1 seems to modulate cell death and not inflammation.

**Figure 2:**
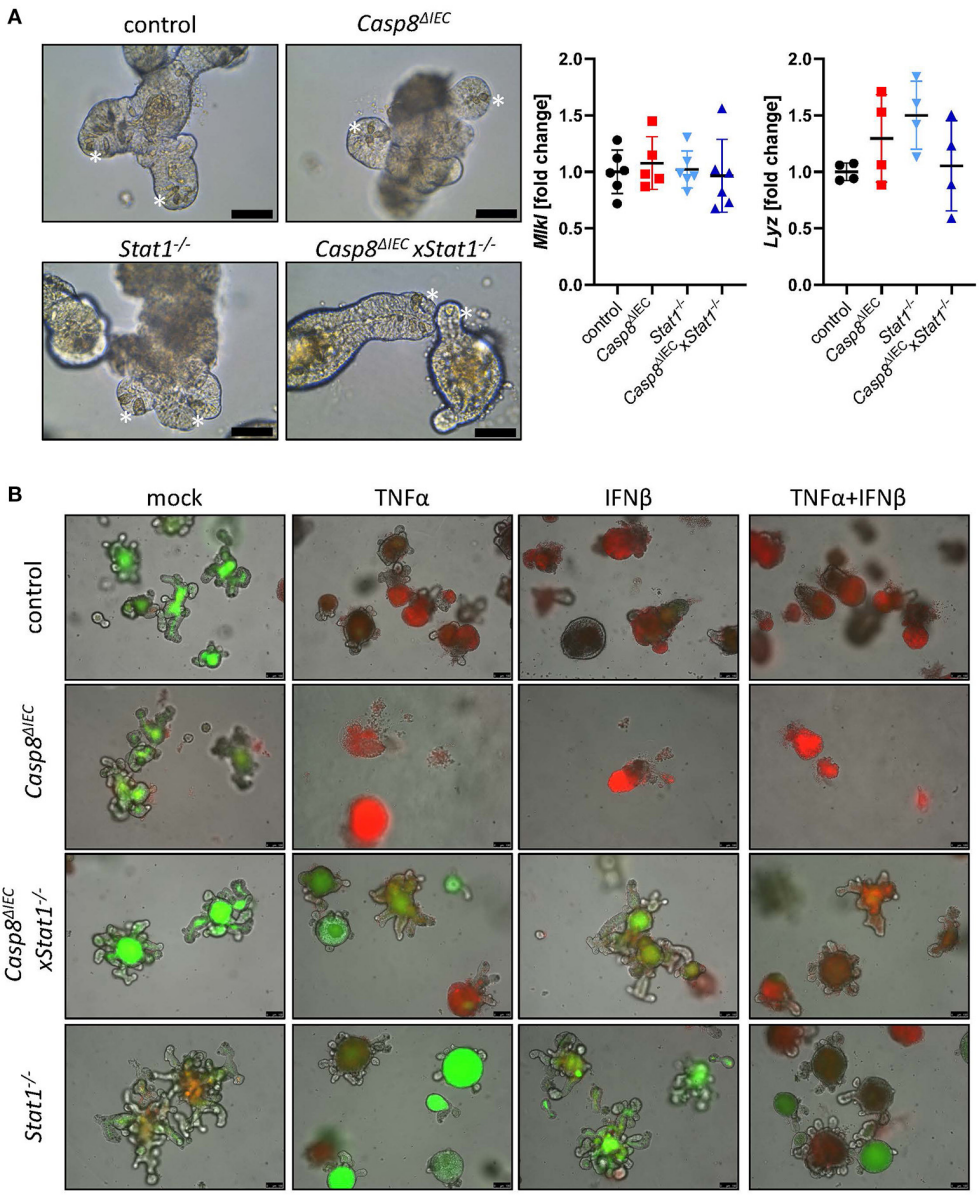
STAT1 coordinates interferon induced cell death *in vitro*. **(A)** Representative pictures of organoids derived from of control, *Casp8*^Δ*IEC*^, Stat1^−/−^ and *Casp8*^Δ*IEC*^xStat1^−/−^ mice. (Scale bar: 50 μm, Paneth cells marked with asterisks). Gene transcription analysis of organoid mRNA expression. *Gapdh* was used as housekeeping gene. Gene expression levels are shown as fold changes. Error bars indicate +/-SD. **(B)** Organoids derived from of control, *Casp8*^Δ*IEC*^, Stat1^−/−^ and *Casp8*^Δ*IEC*^xStat1^−/−^ mice. Stimulated with TNFα (25 ng/ml) and IFNβ (100 ng/ml) were stained with propidium iodide (red, dead cells) and live cells visualized by autofluorescence (green; scale bar: 100 μm).

To further investigate the molecular mechanism underlying epithelial cell death, we took advantage of organoids derived from the small intestine of control, *Stat1*^−/−^, *Casp8*^Δ*IEC*^ and *Casp8*^Δ*IEC*^x*Stat1*^−/−^ double deficient mice. Surprisingly, in contrast to the reduced number of Paneth cells observed *in vivo*, organoids derived from small intestinal tissue of *Casp8*^Δ*IEC*^ and *Casp8*^Δ*IEC*^x*Stat1*^−/−^ mice displayed Paneth cells in an equal number and morphology as in control organoids (Figure 2A, marked by asterisk). In addition, expression of the Paneth cell marker Lysozyme (Lyz) was similar between all organoid cultures (Figure 2A). Interestingly, in contrast to our *in vivo* data, *Mlkl* expression was not elevated in any analyzed group, suggesting that activation of *Mlkl* gene transcription via STAT1 requires IFNs derived from non-epithelial cells or that factors, triggering autocrine IFN production by intestinal epithelial cells, are missing *in vitro*.

*In vivo*, cell death in intestinal epithelial cells can be induced by TNFα or IFNs (13, 17, 34). Interferons are able to induce the expression of *Mlkl*, whereas TNFα act as cell death trigger (13). To simplify the complex *in vivo* situation, we stimulated organoids with IFNβ, an interferon that can induce canonical and non-canonical STAT pathways (4), alone and in combination with TNFα. In line with previous studies, intestinal organoids lacking only *Caspase-8* were highly susceptible in response to TNFα and INFβ (Figure 2B). Additional deletion of *Stat1* was sufficient to block cell death with enhanced viability compared to control organoids (Figure 2B). These results highlight that beside factors that activate cell death, also the expression of cell death mediators such as MLKL, are relevant for cell death induction. However, when organoids were stimulated with TNFα in combination with IFNβ, to mimic a more complex *in vivo* situation, also double deficient organoids started to die, suggesting that the synergistic action of these two cytokines can trigger alternative cell death pathways which are independent of Caspase-8 or MLKL.

In summary these data suggest, that STAT1 is a key transcription factor for *Mlkl* gene expression in intestinal epithelial cells that orchestrate cell death in response to either TNF or IFNs.

### STAT1 Does Not Modulate Cell Death During Experimental Colitis

**Figure 3:**
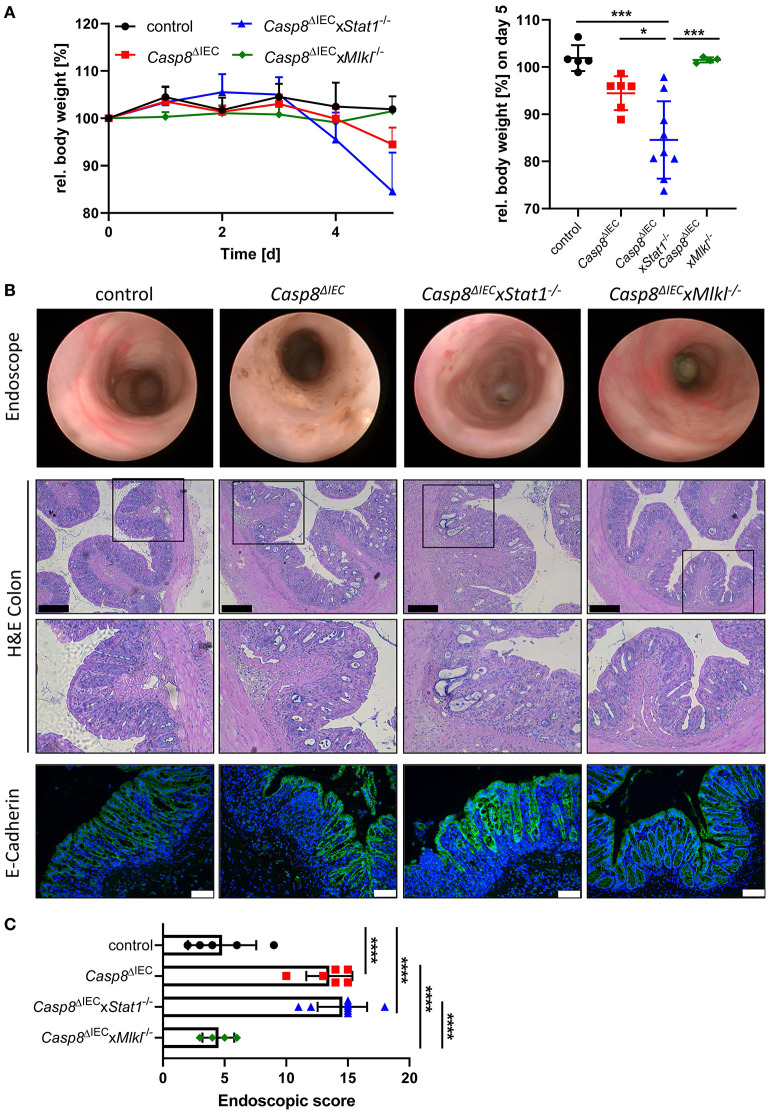
Deletion of STAT1 does not ameliorate inflammation of *Casp8*^Δ*IEC*^ mice during DSS colitis. **(A)** Relative body weight of control, *Casp8*^Δ*IEC*^, *Casp8*^Δ*IEC*^xStat1^−/−^, *Casp8*^Δ*IEC*^x*Mlkl*^−/−^ mice after administration of 2% DSS in drinking water. Mice were sacrificed at day 5. **(B)** Representative endoscopic pictures at day 5. Representative images of colonic cross sections stained with H&E (scale bar: 200 μm) or immunohistochemically stained (scale bar: 75 μm) with antibody against E-Cahderin (green). Nuclei were counterstained with Hoechst 33,342 (blue). **(C)** Endoscopic score. Error bars indicate +/-SD. Statistical analyses: One-way ANOVA with Tukey's multiple comparisons test; NS *p* ≥ 0.05; * *p* < 0.05; **p < 0.01; ***p < 0.001; ****p < 0.0001. Pooled data of two individual experiments.

Emerging evidence suggests that enterocyte death in the small and the large intestine is mediated by distinct molecular mechanism. Therefore, in a next set of experiments, we aimed to delineate the impact of STAT1 signaling during inflammation-induced cell death in the colon (Figure 3). As previously demonstrated, *Casp8*^Δ*IEC*^ mice only develop colonic inflammation under specific microbial settings or in response to experimental colitis (18). Accordingly, histological analysis of caecal and colonic tissue revealed no signs of inflammation or cell death in all analyzed mouse strains under steady state conditions (control, *Casp8*^Δ*IEC*^, and *Casp8*^Δ*IEC*^x*Stat1*^−/−^ mice) (Supplementary Figure 2). Hence, we decided to trigger colonic inflammation by administration of low-dose DSS in the drinking water. Surprisingly, in sharp contrast to our *in vitro* observation and *in vivo* data derived from the small intestine, STAT1 was not essential to orchestrate necrotic cell death during chemically induced colitis (Figure 3). *Casp8*^Δ*IEC*^ as well as *Casp8*^Δ*IEC*^x*Stat1*^−/−^ mice displayed body weight loss and increased inflammation after DSS administration (Figure 3). Interestingly, body weight loss was most pronounced in *Casp8*^Δ*IEC*^x*Stat1*^−/−^ mice (Figure 3A), while endoscopic analysis and scoring revealed that both groups (*Casp8*^Δ*IEC*^ as well as *Casp8*^Δ*IEC*^x*Stat1*^−/−^ mice) developed equal colonic inflammation (Figures 3A,B). Furthermore, both genotypes display severe tissue destruction, increased cell death and loss of epithelial integrity as demonstrated by H&E- and E-Cadherin staining (Figure 3B). In contrast to the minor contribution of *Stat1* to inflammation induced cell death in the colon, deletion of *Mlkl* was sufficient to rescue *Casp8*^Δ*IEC*^ mice from massive epithelial cell death and associated tissue destruction. Accordingly, *Casp8*^Δ*IEC*^x*Mlkl*^−/−^ mice displayed a similar disease activity and tissue damage compared to wild type littermates (Figure 3). These results suggest that STAT1 acts upstream of MLKL during small intestinal inflammation, but seems to have differential regulatory functions in the large intestine during DSS-induced colitis.

### STAT2 Does Not Prevent Paneth Cell Death

**Figure 4:**
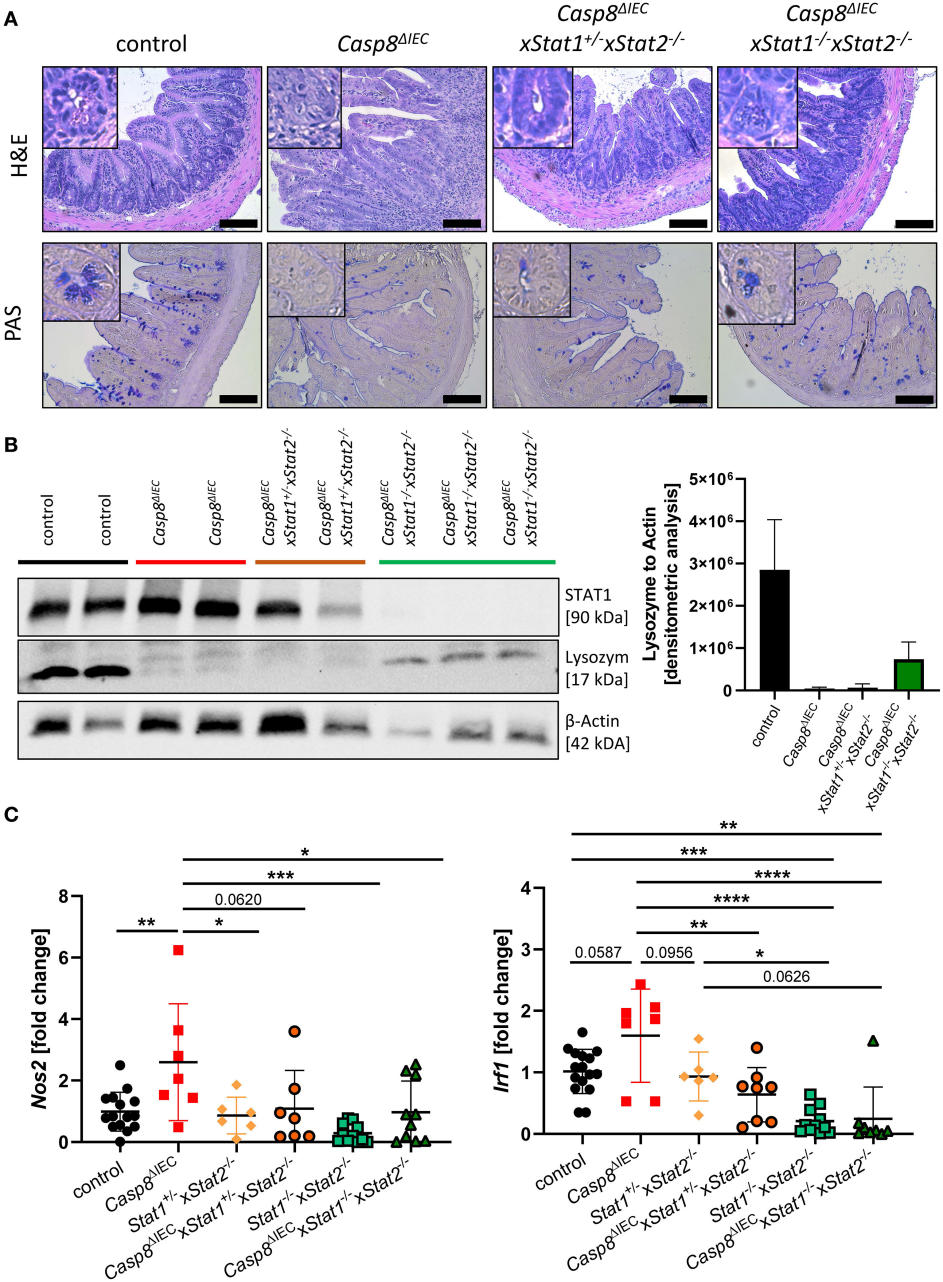
STAT2 signaling fails to restore Paneth cell viability but alters inflammation. **(A)** Representative images of small intestinal tissue cross sections of control, *Casp8*^Δ*IEC*^, *Casp8*^Δ*IEC*^x*Stat1*^+^^/−^*Stat2*^−/−^ and *Casp8*^Δ*IEC*^x*Stat1*^−^^/−^*Stat2*^−/−^ mice stained with H&E and PAS (scale bar: 100 μm). **(B)** Western blot analysis and normalization of ileal tissue with antibodies against STAT1 and Lysozyme. β-Actin was used as loading control. Densitometry analysis for quantification (*n* ≥ 2). Error bars indicate +SD. **(C)** Gene transcription analysis of *Mlkl* mRNA expression in the small intestine. *Gapdh* was used as housekeeping gene. Gene expression levels are shown as fold changes. Error bars indicate +/-SD. Statistical analyses: One-way ANOVA with Tukey's multiple comparisons test; NS *p* ≥ 0.05; *p < 0.05; **p < 0.01; ***p < 0.001; ****p < 0.0001.

IFNs are able to signal through various combination of STATs in response to canonical and non-canonical JAK-STAT signaling (4). To investigate if an additional pathway, mediated by STAT2, might influence cell death in the intestinal epithelium, we generated *Caspase-8* and *Stat2* double deficient mice (*Casp8*^Δ*IEC*^x *Stat1*^+^^/−^*Stat2*^−/−^ mice) as well as triple knockout animals lacking both STAT1 and STAT2 members (*Casp8*^Δ*IEC*^x *Stat1*^−/−^x*Stat2*^−/−^). Similar to organoids derived from *Casp8*^Δ*IEC*^x*Stat1*^−/−^ mice, Paneth cell numbers and expression of antimicrobial peptides was not influenced by additional deletion of *Stat2* in *Casp8*^Δ*IEC*^ mice *in vitro* (Supplementary Figure 3). However, in contrast to *Casp8*^Δ*IEC*^x*Stat1*^−/−^ mice, *Stat2* deficiency did not improve Paneth cell viability *in vivo* as we observed only rare numbers of these secretory cells at the crypt bottom (Figure 4A). These histological features were supported by quantification of Lysozyme by Western Blot, which revealed similar levels between *Casp8*^Δ*IEC*^ and double deficient mice (*Casp8*^Δ*IEC*^x *Stat1*^+^^/−^*Stat2*^−/−^ mice) (Figure 4B). In line with these data, we identified high numbers of TUNEL positive cell along the crypt-villus axis of *Stat2* deficient *Casp8*^Δ*IEC*^ mice (Supplementary Figure 4A). In contrast to this, *Mlkl* gene expression was downregulated in all groups lacking a single STAT member or both compared to *Casp8*^Δ*IEC*^ mice (Supplementary Figure 4B), suggesting that alternative cell death pathways are activated.

### STAT2 Signaling Contributes to Small Intestinal Inflammation

While our data suggested a neglectable function of STAT2 in coordinating cell death in this mouse model of ileitis, we surprisingly observed that STAT2 influences small intestinal inflammation. Accordingly, as previously described *Casp8*^Δ*IEC*^ mice display severe inflammation including bowel wall thickening and an increased cellularity in the lamina propria of the terminal ileum. By contrast, deletion of STAT2 in these mice decreased these histo-morphological features (Figure 4A). In line with these results, gene expression of *S100a9* as well as *Nos2*, both associated with intestinal inflammation and upregulated in *Casp8*^Δ*IEC*^ mice, were downregulated in *Casp8*^Δ*IEC*^x *Stat1*^−^^/−^*Stat2*^−/−^ mice triple deficient mice and reduced in *Casp8*^Δ*IEC*^x *Stat1*^+^^/−^*Stat2*^−/−^ double deficient mice (Figure 4C, Supplementary Figure 4C). Moreover, *Irf1*, a downstream target of IFNγ-STAT1 signaling was significantly reduced in *Casp8*^Δ*IEC*^x *Stat1*^+^^/−^*Stat2*^−/−^ mice (Figure 4C).

In summary, we provide molecular evidence that STAT2 alone or in combination with STAT1 contributes to small intestinal inflammation. Our results further demonstrate that STATs individually affect the distinct pathophysiology of IBD in the ileum and colon, respectively, which might explain the diverse outcome of JAK inhibitors in IBD patients with different localization of the inflammation site.

## Discussion

The pathogenic mechanisms involved in IBD contain a complex network of several key factors including immune cells, cytokines and the intestinal epithelial barrier. Breakdown of the intestinal epithelial barrier, caused by massive cell death or missing mucosal healing is a crucial step during IBD pathology, which consequently also influences immune responses and microbial composition by missing antimicrobial defense (35). Moreover, clinical research and studies in experimental disease models have delineated the ambivalent role of IFNs and STAT1 in orchestrating epithelial cell homeostasis including induction of death as a key aspect of chronic inflammation as well as conducting mucosal healing during colonic inflammation (13, 14). Accordingly, blocking IFN signaling is a promising novel therapy for patients suffering from IBD. However, the underlying molecular mechanism and targeted cells are still controversially discussed.

Here, we provided novel evidence that STAT1 and STAT2 might independently influence intestinal inflammation in a highly spatial-dependent process. Blocking STAT1 signaling by genetic deletion of this transcription factor in *Casp8*^Δ*IEC*^ mice, was sufficient to partially rescue Paneth cell depletion and to reduce cell death frequency the intestinal epithelium (Figure 1). These data are in line with the observation that the IFNλ-STAT1 signaling axis is a key factor in small intestinal inflammation (13). Accordingly, two groups independently demonstrated that IFNs can either directly, or indirectly through IL-22, trigger non-apoptotic cell death (13, 36). Moreover, in a translational approach it has been described that IFNλ might support ileal inflammation by mediating necrotic Paneth cell death coordinated by STAT1 and MLKL (13). Here, we uncovered the fact that STAT1 was not able to fully restore Paneth cell viability *in vivo*, suggesting that further factors, triggering cell death, or additional pathways are present in the context of CD manifestations like ileitis. *In vitro* experiments using organoids, demonstrated that epithelial cells lacking Caspase-8 and STAT1 were protected from TNF or IFN induced toxicity, while single knock-out organoids displayed excessive cell death. These data demonstrate that both factors are sufficient to trigger cell death, but that *in vivo* additional factors are present that might activate Paneth cell necroptosis. Interestingly a recent paper identified the Z-DNA-binding protein 1 (ZBP1) as potential novel player in the pathogenesis of intestinal inflammation. ZBP1, also known as DAI, was initially identified to induce IFN-mediated MLKL-dependent necroptosis in the context of viral infection (37, 38). However, recent studies in mice and humans, further unveiled its contribution to gastrointestinal inflammation (19, 39). Interestingly, genome instability in IBD patients could trigger ZBP1 activation associated with necroptosis. Murine genomic instability, mimicking the human situation, was associated with ZBP1 activation, MLKL-mediated necroptosis and followed disruption of the epithelial barrier (39).

Beside their impact on cell death regulation, IFNs are primarily known for their immune-modulatory function. Accordingly, while STAT1 was sufficient to block TNFα or IFN triggered cell death *in vitro* and partially rescued Paneth cell death *in vivo*, it was surprisingly not involved in inflammation in the small intestine. Moreover, we identified that STAT1 signaling might be associated with tissue injury processes in the colon as *Casp8*^Δ*IEC*^x *Stat1*^−^^/−^ mice exhibited severe tissue injury and inflammation in response to experimental colitis. These data are in line with a previous publication by Chiriac et al., highlighting that the activation of IFNλ-STAT1 signaling specifically in the IECs is responsible for mucosal healing and epithelial regeneration during colitis (14). Our results highlights differential mechanisms and upstream regulatory components underlying cell death pathways in the small and large intestine. In sharp contrast to these results, deletion of STAT2 in *Casp8*^Δ*IEC*^ mice, was associated with mucosal healing and reduction of disease activity in the small intestine, while Paneth cell homeostasis was not influenced by STAT2. STAT2 is linked to type I interferon signaling and only little is known about its role during IBD (28). In humans, downregulation of STAT2 gene expression has been observed in LPMCs (lamina propria mononuclear cells) derived from IBD patients (28, 40). Further studies are required to address the role of STAT2 in the context of human and murine intestinal inflammation.

Our findings on the differential role of STAT signaling molecules in the context on ileitis and colitis are in line with previous studies, supporting the concept that the pathogenic mechanism underlying ileal and colonic Crohn's disease are distinct and thus require individual therapies. Our data now provide further mechanistic insights in the role and contribution of the JAK-STAT signaling in the intestinal tract, which is currently in clinical focus. Accordingly, blocking JAK-STAT signaling is a promising therapeutic intervention, but current studies uncovered differential therapeutic success between ulcerative colitis and Crohn's disease (22–26). The broad JAK inhibitor tofacitinib (JAK1 and JAK3 inhibitor) showed promising results for patients with ulcerative colitis but not for Crohn's disease. Beside this, more selective inhibitors like filgotinib and upadacitinib (JAK1 inhibitors) seem to have more therapeutic benefit in both diseases (27). In this context, a recent study investigated the impact of both, selective and broad JAK-inhibitors, on ileitis and uncovered that blocking JAK-STAT signaling inhibited Paneth cell dysfunction and inflammation *in vitro* and *in vivo* (13). In line with these previous results, our data also suggest a major contribution of STAT signaling to small intestinal inflammation but not colitis. The fact that STAT signaling influences homeostasis of the intestinal epithelium, as a key component in the pathogenesis of inflammation, in a highly regional manner, indicates that further studies are required to fully define the contribution of STAT1 and STAT2 to inflammatory processes in the small and large intestine.

In summary, we provide molecular evidence that STAT1 and STAT2 both contribute to intestinal inflammation but have differential functions. Our results demonstrate that STAT1 coordinates cell death in the ileum but not during experimental colitis. Furthermore, STAT2 was able to modulate mucosal inflammation, independent of STAT1. Thus, our data provide further evidence for a differential pathological mechanism responsible for ileal and colonic inflammation.

## Data Availability Statement

The raw data supporting the conclusions of this article will be made available by the authors, without undue reservation.

## Ethics Statement

The animal study was reviewed and approved by Institutional Animal Care and Use Committee of the Regierung von Unterfranken and conducted by qualified personnel.

## Author Contributions

IS, MN, and CG: designed the research. IS and AD: performed the experiments. MC: supplied material. IS, MN, and CG: analyzed the data and wrote the paper. All authors contributed to the article and approved the submitted version.

## Conflict of Interest

The authors declare that the research was conducted in the absence of any commercial or financial relationships that could be construed as a potential conflict of interest.
