# Supplementary material for: An IFN-STAT Axis Augments Tissue Damage and Inflammation in a Mouse Model of Crohn's Disease
Source: Front Med (Lausanne). 2021 May 20;8:644244. doi: 10.3389/fmed.2021.644244 (PMC8205542; doi:10.3389/fmed.2021.644244)
Supplement: Supplementary file 1 [file Data_Sheet_1.docx]

Supplementary Material

**Supplementary Figure legend**

Figure S1: Paneth cell depletion is mediated by MLKL

Representative images of small intestinal tissue cross sections of control, *Casp8*^ΔIEC^, and *Casp8*^ΔIEC^x*Mlkl*^-/-^ mice stained with H&E (upper panel) or PAS (lower panel) (scale bar: 100 μm).

Figure S2: No histo-morphological alterations in cecum and colon

**A)** Representative images of small intestinal tissue cross sections of control, *Casp8*^ΔIEC^, *Casp8*^ΔIEC^x*Stat1^-^*^/-^ mice stained with antibody against CD45 (red; scale bar: 50 μm). Nuclei were counterstained with Hoechst 33342 (blue). **B)** Representative images of cecal and colonic cross sections of control, *Casp8*^ΔIEC^, and *Casp8*^ΔIEC^x*Stat1*^-/-^ mice stained with H&E (upper panel) or PAS (lower panel) (scale bar: 100 μm).

Figure S3: Restored Paneth cell viability *in vitro*

Representative pictures of organoids derived from of control, *Casp8*^ΔIEC^, *Stat1*^-/-^ and *Casp8*^ΔIEC^x*Stat1*^-/-^ mice. (Scale bar: 50 μm, Paneth cells marked with asterisks). Gene transcription analysis of organoid mRNA expression. *Gapdh* was used as housekeeping gene. Gene expression levels are shown as fold changes. Error bars indicate +/-SD.

Figure S4: STAT2 does not control cell death

**A)** Representative images of small intestinal tissue cross sections of control, *Casp8*^ΔIEC^x*Stat1^+^*^/-^*Stat2*^-/-^ mice stained with TUNEL assay (red; scale bar: 100 μm). Nuclei were counterstained with Hoechst 33342 (blue). **B-C)** Gene transcription analysis of mRNA expression derived from the small intestinal tissue of wild type control, *Casp8*^ΔIEC^, *Casp8*^ΔIEC^x*Stat1^+^*^/-^*Stat2*^-/-^, *Stat1^+^*^/-^*Stat2*^-/-^, *Casp8*^ΔIEC^x*Stat1^-^*^/-^*Stat2*^-/-^, *Stat1^-^*^/-^*Stat2*^-/-^ mice. *Gapdh* was used as housekeeping gene. Gene expression levels are shown as fold changes. Error bars indicate +/-SD. Statistical analyses: One-way ANOVA with Tukey's multiple comparisons test; NS p ≥ 0.05; * p < 0.05; ** p < 0.01; *** p < 0.001; **** p < 0.0001.

**Supplementary table**

**Table 1**

| **Antiboy** | **Cat. No.** | **Manufacture** | **Application** |
| --- | --- | --- | --- |
| Anti-Lysozyme antibody | ab108508 | abcam | WB |
| Anti-Lysozyme antibody | A0099 | Dako | IHC |
| Anti-rabbit IgG, HRP-linked Antibody | 7074 | Cell Signaling | WB |
| Anti-Stat1 Antibody | 9172 | Cell Signaling | WB |
| Biotin Goat Anti-Rat Ig | 554014 | BD Pharmingen | IHC |
| Biotin Goat Anti-Rat Ig | 554014 | BD Pharmingen | IHC |
| Biotin-SP Goat Anti-Rabbit IgG | 111-065-144 | Jackson ImmunoResearch | IHC |
| CD45 Antibody (30-F11) | 14-0451-82 | eBioscience | IHC |
| FITC Mouse Anti- E-Cadherin | 612130 | BD Pharmingen | IHC |

**Table 2**

| **Gene** | **QuantiTect Primer Assay** | **Cat. No.** |
| --- | --- | --- |
| *S100a9* | Mm_S100a9_1_SG | QT00105252 |
| *Mlkl* | Mm_Mlkl_1_SG | QT01069285 |
| *Irf1* | Mm_Irf1_1_SG | QT00128989 |
| *Lyz* | Mm_Lyz1_1_SG | QT00143269 |
| *Nos2* | Mm_Nos2_1_SG | QT00100275 |
| ***Gene*** | **Sequence** |  |
| *Gapdh* | TCACCACCATGGAGAAGGC  GCTAAGCAGTTGGTGGTGCA |  |
