# Supplementary figures and images for: An IFN-STAT Axis Augments Tissue Damage and Inflammation in a Mouse Model of Crohn's Disease
Source: Front Med (Lausanne). 2021 May 20;8:644244. doi: 10.3389/fmed.2021.644244 (PMC8205542; doi:10.3389/fmed.2021.644244)

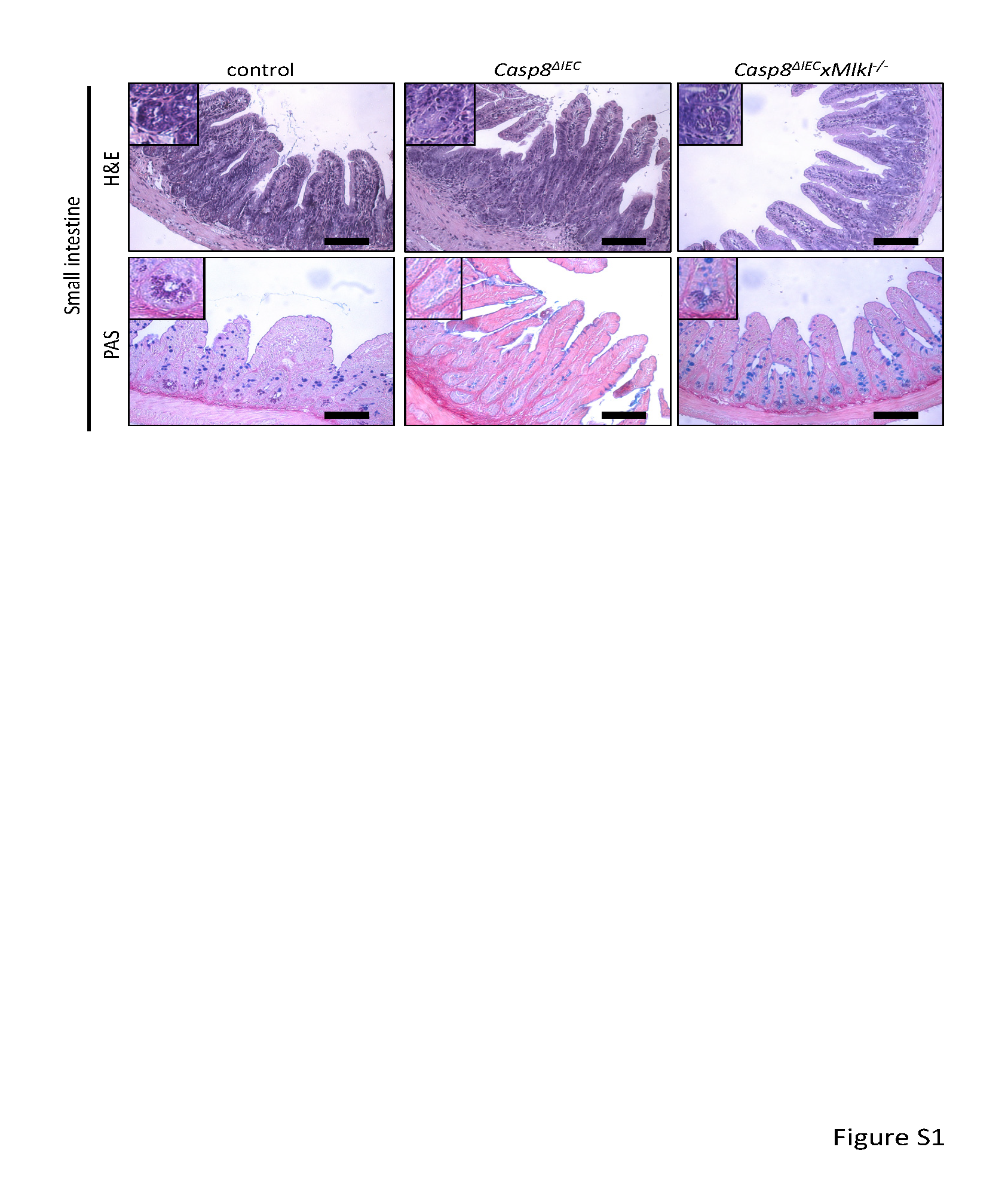

Supplement: Supplementary file 2 [file Image_1.TIFF]

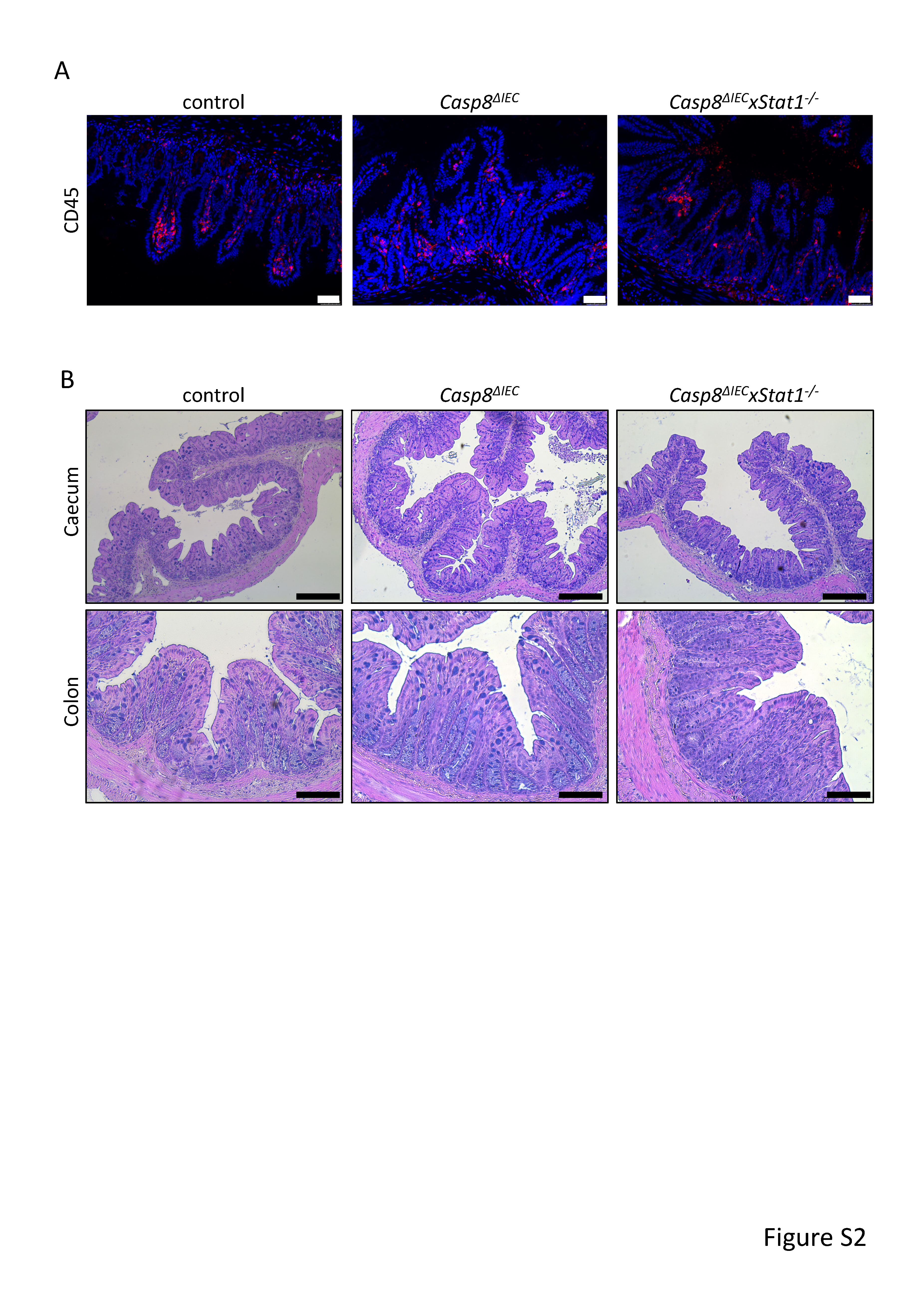

Supplement: Supplementary file 3 [file Image_2.TIF]

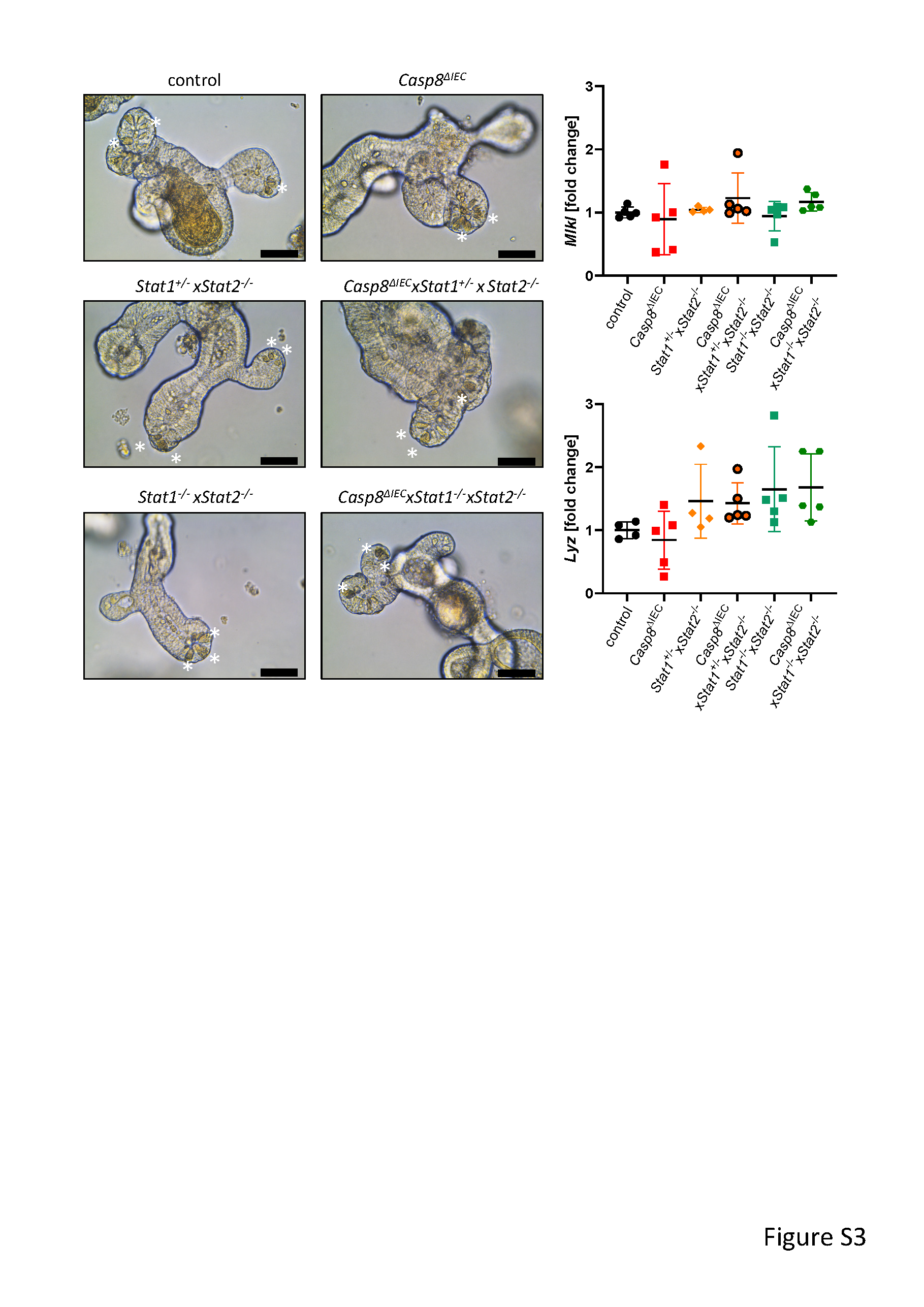

Supplement: Supplementary file 4 [file Image_3.TIFF]

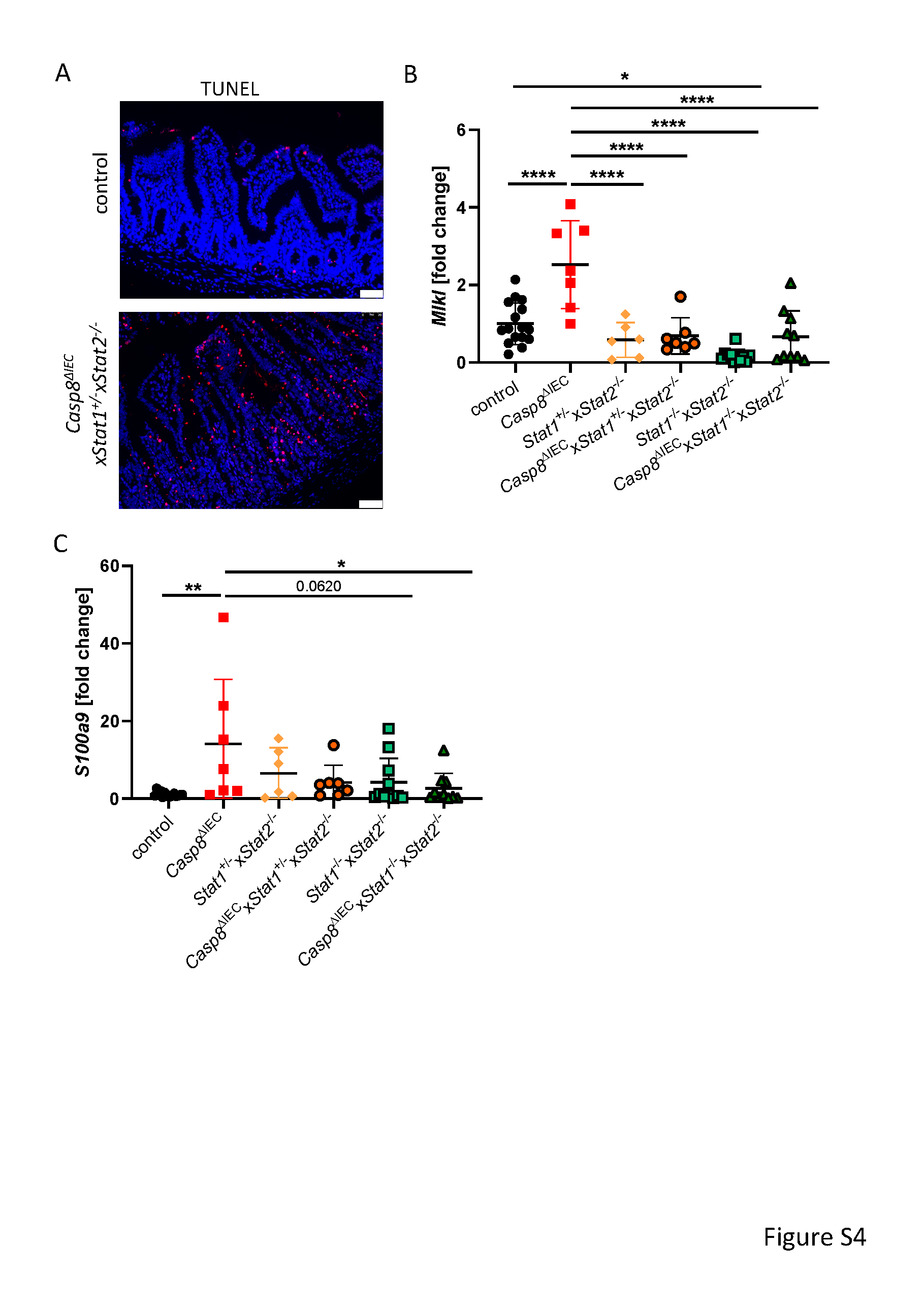

Supplement: Supplementary file 5 [file Image_4.TIFF]
